# Cheoplasty

**Published:** 1858-04

**Authors:** 


### CHEOPLASTY.

In a previous issue, I endeavored to explain my position on the subject, but from numerous letters received, it does not seem to be fully understood.

I am not disposed at present, either to recommend or denounce the method. Sufficient time has not elapsed for many to form definite conclusions. From the testimony of those who have been using it, we have no doubt that under ordinary circumstances, it will stand the test so far as destructibility is concerned.

The work can be made as perfect in its adaptation as any other style. The metal is perfectly adapted to the teeth, and no interstices are left to afford lodgment for food or any foreign substance. Any desired form may be given to the plate for the comfort and convenience of contiguous parts. In the most of the early experiments, an error was committed in making the plates too heavy and clumsy—supposing this to be necessary to obtain sufficient strength, the thickness required is from 24 to 26 Stubs guage, slightly thickening towards the teeth.

Drs. Oliver & Harrison, of Buffalo, N. Y., have a patent now thrown open to the Profession, dated May 20th, 1856, for teeth and a process in some respects similar. And they claim that Blandy's process is an infringement on theirs. We are frequently asked if the metal we sell is an *infringement* on Blandy's. Whether we will guarantee those who use it from prosecution, and whether we will defend them in case of prosecution. To each of these questions, we answer emphatically, NO.

The metal is not an infringement. But we can not guarantee that those who use it may not be prosecuted; because they might be sued without any just cause.

We could not defend them, because we have neither time nor money to spend traveling all over the country, from Court-House to Court-House, to defend suits in which we have neither profit nor interest.

We have never urged any one to use it, we do not care whether they do or not, and would not give advice to our friends, which could by any possible chance lead them into difficulty; we can only place the knowledge and means within your reach, and each one must judge for himself *how to make use of the knowledge.* We have spent some $200, (more by half than we ever expect to realize from it), in placing this knowledge and means before the profession, and we think we have done our whole duty, for the rest they must use their own judgment.

If any wish to investigate the matter further for themselves, they can by enclosing one dollar to Commissioner of Patents, obtain a copy of the Patent itself. We think, Dr. Blandy has committed an error, and we are sorry for it, as he seems from our intercourse with him, to be a very clever gentleman—as all who have attempted Dental Patents—in placing restrictions on the *right to use*; if he had confined himself to the sale of the teeth, metal, &c., at a reasonable price, nine-tenths of the Profession would have taken it for *experiment at least.*

If this process has the merit of originality and usefulness, there is no cause for hiding it in mystery, for truth, justice, and merit, will increase and gather strength in proportion as they are exposed.

We are perfectly prepared to defend ourselves from any assault that may be made, we have infringed no laws, nor invaded any man's rights, but simply performed our duty to the profession, and this we intend ever to do; if this or any other process will not bear the flaming light of *truth*, then let it go to the shades—the sooner the better.

